# Investigating the Effects of Coenzyme Q10 on Human Corneal Endothelial Cells

**DOI:** 10.1155/2021/8392572

**Published:** 2021-08-12

**Authors:** Mitchell Titley, Sajjad Ahmad, Mohit Parekh

**Affiliations:** ^1^Institute of Ageing and Chronic Disease, University of Liverpool, Liverpool, UK; ^2^Institute of Ophthalmology, University College London, London, UK; ^3^Moorfields Eye Hospital NHS Trust Foundation, London, UK

## Abstract

**Purpose:**

To investigate the effects of Coenzyme Q10 (CoQ10) treatment on immortalised human corneal endothelial cells (HCEC-12).

**Methods:**

HCEC-12 cells were cultured in different concentrations of CoQ10 (0.1%, 0.2%, 0.5%, and 1.0%) and analysed using live/dead staining assay to determine appropriate concentration for subsequent experiments. Cells were pretreated with CoQ10 before inducing apoptosis by ethanol (EtOH) treatment for 30 seconds which was followed by posttreatment with CoQ10. Viable, apoptotic, and dead cell proportions were analysed using Annexin V-FITC immunofluorescence staining. Mitochondrial intensity and respiratory functions were also investigated using MitoTracker staining and a Seahorse XFe24 analyser, respectively. Results were compared to a positive control for apoptosis. The experiments were carried out in triplicates. Graphpad prism software was used for statistical analysis where *p* < 0.05 was deemed statistically significant.

**Results:**

CoQ10 treatment at 0.5% and 1% showed 92% and 30% viable cells compared with 0.1% and 0.2% that showed 96% and 94% viable cells, respectively (*p*=0.0562). 0.1% and 0.2% concentrations were, thus, used for subsequent experiments. Annexin V-FITC apoptotic analysis showed 2% at 0.1% and 3% at 0.2% of apoptotic cells (*p*=0.0824). Mitochondrial respiratory function and mitochondrial intensity increased in apoptotic cells following 0.1% CoQ10 treatment.

**Conclusion:**

0.1% CoQ10 was found optimal for reducing apoptosis and increasing metabolic activity on human corneal endothelial cell line. These results support the need for further *ex vivo* studies to investigate the safety profile of CoQ10 as an antiapoptotic agent for human corneal endothelium.

## 1. Introduction

The human cornea is an avascular tissue at the front of the eye that has a posterior monolayer of mitochondria-rich hexagonal cells known as ‘endothelial cells.' The endothelial cells are responsible for regulating water and mineral transport to the entire cornea, whilst maintaining a relatively dehydrated corneal state (deturgescence) for transparency [1] using a process known as “pump-leak hypothesis” [2, 3]. Corneal endothelial diseases are an important cause of worldwide blindness. Many endothelial disease processes can result in visual loss, including Fuchs' dystrophy [4], posterior polymorphous dystrophy, and pseudophakic bullous keratopathy [5]. Although the disease processes inducing apoptosis and/or cell death are found to be multifactorial, it has been suggested that mitochondrial oxidative dysfunction may play a role in these metabolically active cells [3, 6]. Corneal transplants (including selective corneal layer keratoplasty, such as Descemet's stripping automated endothelial keratoplasty (DSAEK) and Descemet's membrane endothelial keratoplasty (DMEK)) are currently the definitive treatment options for endothelial disease [7]. There is a worldwide shortage of donor corneas, and there are risks associated with corneal transplant surgery, including infection, tissue rejection, or failure [8, 9].

Coenzyme Q10 (CoQ10) is a naturally occurring quinone that is predominantly acquired through dietary sources but is also synthesised in the liver. The ability of the molecule to form both oxidised and reduced states allows it to support normal cellular function as well as inhibit cell apoptosis through a variety of mechanisms. CoQ10 plays an important role in the maintenance of the proton gradient at complexes I and II of the electron transport chain of the inner mitochondrial membrane, an important process required for oxidative respiration and ATP generation for cellular metabolic requirements [10, 11]. CoQ10 is also able to act as an antioxidant to neutralise reactive oxygen species that presents following metabolic processes and can have harmful effects on cells, including induction of apoptosis [11]. CoQ10 has been shown to inhibit PTP channel opening to further inhibit the induction of apoptosis [12]. Individuals with CoQ10 deficiency disorders exhibit heterogeneous clinical symptoms such as encephalomyopathy, severe infantile multisystemic disease, cerebellar ataxia, isolated myopathy, and nephrotic syndrome as a result of dysfunctional cellular metabolic function [13]. Meta-analyses on the cardiovascular effects of CoQ10 treatment have shown improved clinical outcomes such as improved systolic function during heart failure and decrease in cardiovascular biochemical risk factors (triglycerides and plasma lipoproteins) [14, 15]. Similar study designs on CoQ10 treatments in neurodegenerative diseases such as Parkinson's disease, Huntington's disease, and Friedrich's ataxia have also demonstrated protective effects, with decrease in the rates of disease progression [16]. Both in vivo and in vitro studies on the posterior segment of the eye (including optic nerve head astrocytes and retinal ganglion cells) have shown improved cellular responses to apoptosis-inducing stresses following treatment with CoQ10 [17, 18]. Similarly, in vitro corneal fibroblasts and epithelial cells have also shown reductions in cellular damage following the treatment with CoQ10 [19, 20].

CoQ10 treatment for corneal endothelial disease would provide an alternative, less-invasive definitive treatment option to corneal transplants as well as prevent the occurrence of cellular apoptosis [21] in at-risk groups (such as patients undergoing cataract surgery) if given prophylactically. Hence, we set out to investigate the antiapoptotic effect of CoQ10 on human corneal endothelial cell line (HCEC-12) to understand its role in the future therapeutic options to treat, prolong the occurrence, or prevent endothelial cell apoptosis.

## 2. Materials and Methods

### 2.1. Ethical Approval

This project received approval by the University College London ethics committee. No external ethical review was required (under the Human Tissue Act 2004 or Health Research Authority guidance) as human cell lines were used. This project was performed in accordance with the tenets of the Declaration of Helsinki.

### 2.2. Cell Culture

An immortalised human corneal endothelial cell line (HCEC-12) was used for cell culture. HCEC-12 culture media consisted of 95% of a 1 : 1 ratio of Ham's F12 and Medium 199 and 5% foetal bovine serum (FBS). Cells were cultured to form a cell monolayer in T75 flasks and incubated at 37°C, 5% CO_2_, and 95% humidity until full cell confluence. Trypsinisation at 37°C, 5% CO_2_, and 95% humidity for 3–5 minutes was used to detach the cell monolayer and centrifuged at 1000 rpm for 5 minutes to form a pellet of HCECs for passaging cells as well as seeding cells at specified densities for other experiments.

### 2.3. CoQ10 Concentration Titration

Cells were initially cultured in normal HCEC-12 media on 4-well chamber slides until reaching around 80% cell confluence. Normal HCEC-12 media were then replaced with CoQ10 (Sigma-Aldrich, St. Louis, Missouri, USA)-containing HCEC-12 media of different concentrations (0.1%, 0.2%, 0.5%, and 1.0%) and left to incubate for 24 hours. Higher concentrations (0.5% and 1%) were used to investigate if a higher concentration could have an additional beneficiary or toxic effects on the cells. A positive control condition (demonstrating apoptotic cells) was set up consisting of cells that were treated with 20% ethanol (ETOH) for 30 seconds and cultured in normal HCEC-12 media. A negative control condition (demonstrating nonapoptotic cells) was also set up and consisted of cells which remained cultured in normal HCEC-12 media without any additional treatments.

### 2.4. Live/Dead Staining and Analysis

Cells were cultured in different concentrations of CoQ10 (as above) on 4-well chamber slides and analysed using a viability/cytotoxicity kit (Thermofisher, Waltham, MA, USA) to confirm the optimal concentration of CoQ10 to treat cells. Staining of cells with immunofluorescent dyes involved removing HCEC-12 media, washing the slides in sterile phosphate-buffered saline (PBS), and adding the stains. Calcein AM (0.2%), Ethidium Homodimer-1 (0.4%) (both Invitrogen), and Hoechst (0.2%, Thermo Fisher Scientific) stains were diluted in PBS. 200 *μ*l of the final solution was added on the cells. Cells were left to incubate at room temperature (RT) for 30 minutes, and the solution was then removed. The cells were again washed in PBS, and 10 *μ*l of the mounting medium (without DAPI) was topically applied. The cover slips were placed on the slide. Images of cells were taken using a confocal microscope (LSM-700), and image analysis was completed using ImageJ software. All conditions were measured in triplicate for this experiment.

### 2.5. Annexin V-FITC Apoptosis Assay Using Immunofluorescence

Cells were pretreated with CoQ10-containing media to investigate the inhibition of apoptosis in cells that were treated with EtOH to induce apoptosis. Cells were seeded in 4-well chamber slides and cultured in normal HCEC-12 media until approaching cell confluence. The culture media was replaced with 0.1% or 0.2% CoQ10-containing HCEC-12 media, and cells were cultured in these conditions for a further 24 hours. Cells were then treated with 20% EtOH for 20 seconds to induce apoptosis [20], washed with PBS thrice, and replaced with the same concentrations of CoQ10-containing media for 6 hours. Cells treated with EtOH and replaced with regular HCEC-12 media were used as a positive control for apoptosis, and cells receiving no EtOH treatment and remaining in normal HCEC-12 media were used as a negative control. Cells were then stained for analysis at 6 hours after EtOH treatment using the Annexin V-FITC apoptosis staining kit (Abcam, Cambridge, UK). 500 *μ*l binding buffer (containing 0.8% Apopxin Green, 0.4% Cytocalcein, and 0.4% 7AAD stains) was added on the cultured cells, and the cells were incubated for 30 minutes at RT in darkness until viewed using an LSM-700 confocal microscope. All the conditions were measured in triplicates.

### 2.6. Mitochondrial Oxygen Consumption Rate (OCR) Assay

The Seahorse XFe24 analyser was used to calculate OCR (a measure of mitochondrial respiration) in HCEC-12 cells using the Agilent Seahorse XF Cell Mito Stress Test Kit (Agilent Technologies). Cells were seeded in a 24XF cell culture microplate to reach 150,000 cells/mm^2^ in each well at the point of EtOH treatment. The cells were similarly pretreated for 24 hours with 0.1% and 0.2% CoQ10, followed by treatment with EtOH (20% for 30 seconds to induce apoptosis) and a further 6-hour post-EtOH incubation period with cells in CoQ10-containing media. At 6 hours, CoQ10-containing cell culture media were replaced with 500 *μ*l of the prepared seahorse medium (containing Seahorse XF DMEM media, 2 mM L-glutamine, 10 mM glucose, and 1 mM pyruvate and NaOH (added to maintain a 7.4 pH)) and incubated at 37°C for 30 minutes. Measurements of mitochondrial respiration and glycolysis were carried out as previously described [22]. In brief, cells were treated with 1.0 *μ*M concentrations of oligomycin (ATP synthase inhibitor of complex V), carbonyl cyanide-p-trifluoromethoxyphenylhydrazone (FCCP, electron transport chain (ETC) uncoupler) and rotenone with antimycin A (both ETC inhibitors of complex I and III, respectively) throughout the analysis. These treatments, which were added to the cells at specified time points, allowed for calculations of various mitochondrial respiratory parameters, including baseline OCR, ATP production, maximal respiration, proton leakage, and nonmitochondrial oxygen consumption. All conditions were measured in triplicate (2 biological (3 technical)), and repeats were performed for this experiment.

### 2.7. Mitochondrial Staining Intensity Assay

Cells were seeded at 5,000 cells/mm^2^ in each well of a 4-well chamber slide for mitochondrial intensity staining analysis and cultured in normal HCEC-12 media until cell confluence. The culture media were replaced with 0.1% or 0.2% CoQ10-containing HCEC-12 media, and cells were cultured in these conditions for 24 hours. Cells were then treated with 20% EtOH for 30 seconds to induce apoptosis [20], washed with PBS thrice, and replaced with the same concentrations of CoQ10-containing media for 6 hours. Cells treated with EtOH and replaced with regular HCEC-12 media were used as a positive control for apoptosis, and cells receiving no EtOH treatment and remaining in normal HCEC-12 media were used as a negative control. Cells were then stained for analysis at 6 hours after EtOH treatment. The cells were washed with PBS, and 0.1% MitoTracker Deep Red (ThermoFisher) was added in the well and incubated at RT for 45 minutes in darkness, washed with PBS, and fixed with 4% PFA until viewed and imaged using an LSM-700 microscope. All the conditions were measured in triplicates with 6 images taken per technical repeat, and 5 cells per image were analysed (total of 30 images analysed for each cell condition) for this experiment.

### 2.8. Statistical Analysis

Analysis of normal distribution of data was completed using SPSS software by performing a Shapiro–Wilk test (*p* < 0.05 significance value), a skewness and kurtosis *z*-test, and through visual inspection of normal data histograms, Q-Q plots, and box plots [23, 24]. Statistical significance analysis was completed using Graphpad prism software (version 5); a 1-way ANOVA test with a Tukey post hoc analysis test was performed on parametric data, and a Kruskal–Wallis test with Dunn's post hoc analysis test was performed on nonparametric data.

## 3. Results

### 3.1. Live/Dead Cell Staining Assay Analysis

**Figure 1:**
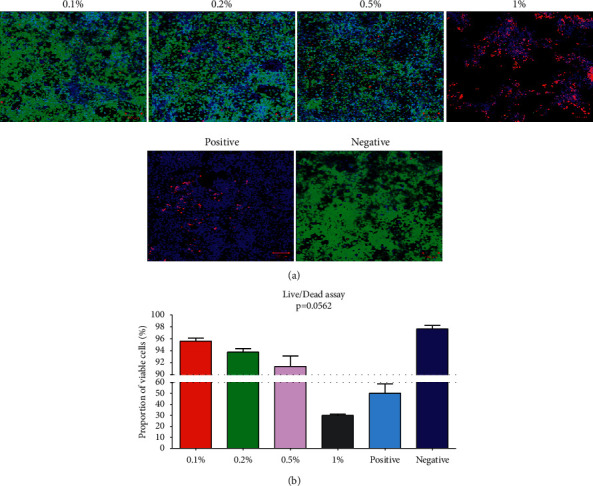
(a) Merged images of live/dead cell staining following cell culture in different concentrations of CoQ10 (Hoechst blue (nuclear), Ethidium homodimer-1 red (dead), and Calcein AM green (viable). (b) Graph showing the results of the mean (and SEM) proportions of viable cells.

The results showed an increasing trend of cell toxicity with increased concentrations of CoQ10 (Figure 1(a)). An average viability of 96 (±0.5)%, 94 (±0.6)%, 92 (±1.7)%, 30 (±0.9)%, 50 (±8.7)%, and 98 (±0.6)% were found in the cells with 0.1%, 0.2%, 0.5%, and 1% positive and negative controls of CoQ10 treatment, respectively, which was not found to be statistically significantly different (*p*=0.0562) (Figure 1(b)).

### 3.2. Annexin V-FITC Apoptosis Assay

**Figure 2:**
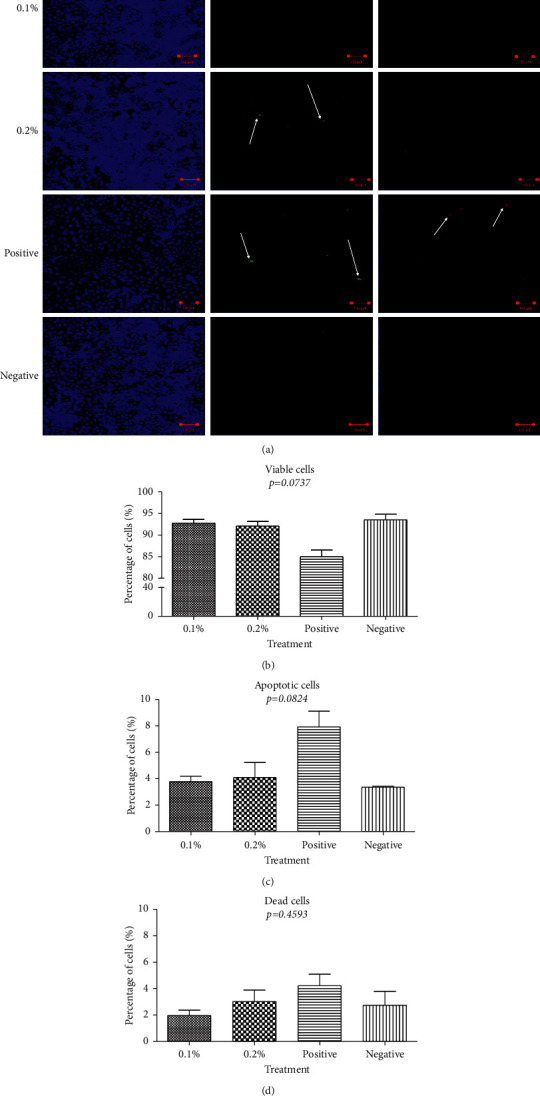
Annexin V-FITC apoptosis assay. (a) Confocal microscopic images of viable cells (blue- cytocalcein), apoptotic cells (green- Apopxin Green: marked with white arrows), and dead cells (red-7AAD: marked with white arrows). (b) Graphical representation of the mean (and SEM) viable, apoptotic, and dead cell proportions through immunofluorescence imaging analysis.

Annexin V-FITC staining showed live, dead, and apoptotic cells from 0.1% and 0.2% positive and negative controls (Figure 2(a)). An average viability of 93 (±1.5)%, 92 (±1.8)%, 85 (±2.6)%, and 93 (±2.2)% (*p*=0.0737) (Figure 2(b)); apoptosis of 2 (±0.7)%, 3 (±1.5)%, 4 (±1.5)%, and 2 (±1.8)% (*p*=0.0824) (Figure 2(c)); and dead cells of 3 (±0.7)%, 4 (±1.9)%, 8 (±2.1)%, and 3 (±0.1)% (*p*=0.4593) (Figure 2(d)) were not found to be statistically significantly different from 0.1% and 0.2% positive and negative controls, respectively.

### 3.3. Mitochondrial Oxygen Consumption Rate (OCR) Assay

**Figure 3:**
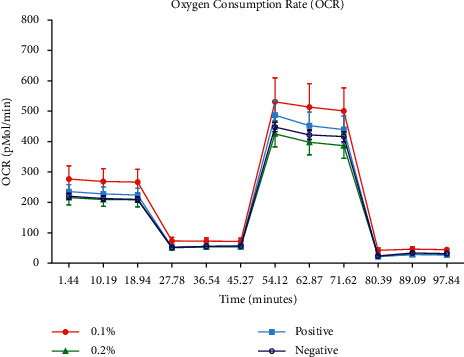
Results of the mitochondrial oxygen consumption rate (OCR) assay and a graph of the Seahorse XF Mito Stress Test profile, showing mean (and SEM) OCR values at given time points.

Mitochondrial OCR, a measure of mitochondrial respiration, was analysed at 6 hours after treatment with EtOH (apoptosis induction) to assess the impact of CoQ10 on mitochondrial function (Figure 3). Similar results were observed between all cell treatments, but a trend showing increased basal OCR, ATP production, nonmitochondrial respiration, maximum respiration, and spare capacity in cells treated with 0.1% CoQ10 was observed.

### 3.4. Mitochondrial Intensity Staining Results

**Figure 4:**
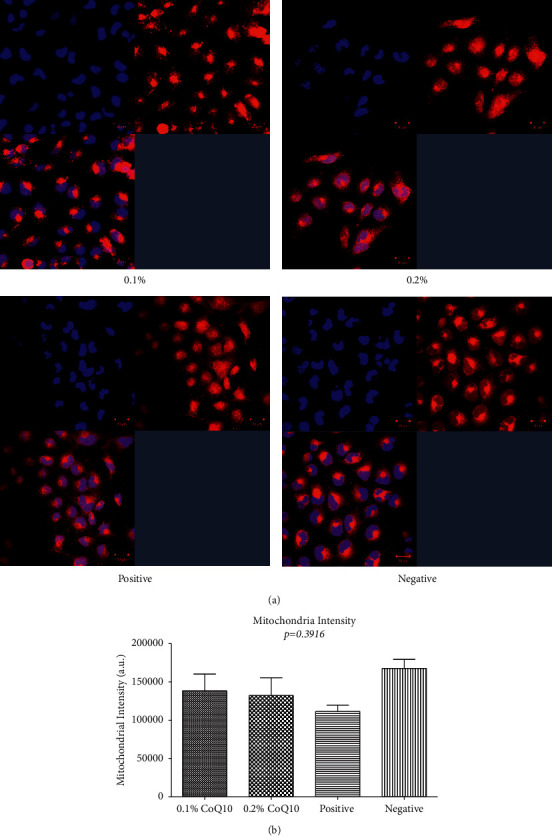
(a) Confocal microscopic images of cells stained with MitoTracker mitochondrial stain at different concentrations of CoQ10. (b) Graphical representation of mitochondrial intensity readings.

Mitochondrial intensity staining was observed using a MitoTracker (Figure 4(a)) in cells at 6 hours after treatment with different concentrations of CoQ10. The results showed a trend of increased mitochondrial intensity in cells treated with CoQ10 as well as in the negative control cells that did not undergo EtOH treatment. Average mitochondrial intensity staining at 0.1% and 0.2%, positive and negative controls, in arbitrary units was 173095 (±50375), 132544 (±22978), 111505 (±8274), and 154936 (±14122), respectively, which was not found to be statistically significant (*p*=0.3916). However, the highest increase in mitochondrial intensity in comparison with positive control samples occurred following 0.1% CoQ10 treatment (Figure 4(b)).

## 4. Discussion

Cytotoxicity with increased concentration of CoQ10 is an important adverse finding from this study. CoQ10 treatment in clinical settings may have significant adverse effects if toxic concentrations are administered or if tissue accumulation of CoQ10 occurs following administration of initially safe concentrations of CoQ10. A previous in vivo study on daily oral and intraperitoneal CoQ10 administration in rats showed liver and spleen accumulation [25]. However, the trends to show the protective effects of CoQ10 in preventing apoptosis and supporting mitochondrial function are in accordance with other previous studies [12, 17].

This study has found that increasing concentrations of CoQ10 (0.5% and 1%) were associated with increased cell death. Lower concentrations (0.1% and 0.2%) were found to be optimal for further ex vivo investigations, as also shown in a previous study [26]. CoQ10 treatment in apoptotic HCEC-12 cells did indicate a trend towards reduced apoptosis and cell death and increased cell viability on immunofluorescence staining analysis. Trends for increased mitochondrial quantity and improved mitochondrial respiratory function were also observed following CoQ10 treatment in apoptotic cells, with 0.1% CoQ10 being shown as the optimal concentration for these improvements.

The results of this study have provided opportunities for other future studies. Investigation into the safety profile of CoQ10 and the mechanisms producing cytotoxicity at increasing concentrations would provide important information and could have wider implications on the systemic treatment capabilities of CoQ10, as well as treatment in corneal endothelial disease. On the other hand, the trends suggesting protective effects of CoQ10 on apoptotic cells mean that similar study designs to further investigate the effects could be applied to ex vivo animal and human primary corneal endothelial cells from donor tissues. These studies would also give rise to opportunities to perform experiments on CoQ10 migration through corneal layers to assess the suitability for a topical eye drop treatment on the corneal surface.

However, limitations to this study undermine the translatability of these results. CoQ10-induced cytotoxicity was shown at higher concentrations in this HCEC-12 cell line, but further experiments are required to assess the optimal/toxic levels of CoQ10 on primary human corneal endothelial cells. Moreover, alongside ethanol treatment, other alternative methods of apoptosis induction could be applied to cells to compare outcomes.

## 5. Conclusions

In conclusion, trends showing CoQ10-associated cytotoxicity at higher concentrations were concerning adverse finding. However, trends showing improved cell viability and decreased cell apoptosis and death in apoptotic cells, as well as trends to show improved mitochondrial quantity and respiratory function at 0.1% concentration, suggest that there are potential benefits of CoQ10 treatment in corneal endothelial disease. These results may also provide grounds for further investigation of CoQ10 as a treatment option for corneal endothelial cell apoptosis.

## Data Availability

Raw data are available in the UCL repository.
